# Case Report: Adult-onset diffuse hepatic hemangiomatosis with rapid progression: an autopsy case with diagnostic difficulty

**DOI:** 10.3389/fmed.2026.1890714

**Published:** 2026-07-21

**Authors:** Takahiro Sugie, Rieko Nishimura, Yuya Urano, Akari Iwakoshi, Yoshiko Murakami, Mariko Sato, Chiyoe Kitagawa, Kenichi Harada

**Affiliations:** 1 Department of Tumor Pathology, Nagoya University Graduate School of Medicine, Nagoya, Japan; 2 Department of Pathology, NHO Nagoya Medical Center, Nagoya, Japan; 3 Department of Medical Oncology, NHO Nagoya Medical Center, Nagoya, Japan; 4 Department of Human Pathology, Graduate School of Medicine Sciences, Kanazawa University, Kanazawa, Japan

**Keywords:** autopsy, diffuse hepatic hemangiomatosis, hepatic small-vessel neoplasm, liver, pathology

## Abstract

Diffuse hepatic hemangiomatosis (DHH) in adults is a rare benign vascular tumor of the liver that histologically resembles a hepatic hemangioma but is characterized by an infiltrative growth pattern despite the absence of cytologic atypia typical of hepatic angiosarcoma. Although generally indolent, its clinical course is variable, and rapidly progressive or fatal cases have been reported. Due to its rarity and overlapping radiological and histopathological features with those of other vascular lesions, its diagnosis can be challenging. We present the case of a 51-year-old female with multiple hepatic lesions, initially suspected to be hepatic hemangiomas. Six months later, the lesions had progressed rapidly. A percutaneous liver biopsy revealed dilated sinusoids lined with endothelial cells without marked atypia, thus suggesting a benign vascular lesion. However, the rapid clinical course raised concerns regarding hepatic angiosarcoma, and the patient received weekly paclitaxel followed by durvalumab. No therapeutic response was observed, and the patient died of tumor rupture. Autopsy revealed diffuse replacement of the hepatic parenchyma with multiloculated cystic vascular spaces lined with pleomorphic endothelial cells. No histological heterogeneity or extrahepatic lesions are observed. These findings supported the diagnosis of DHH. This case report contributes to the current knowledge of the clinical and pathological features of this rare entity. In addition, pleomorphic endothelial cells observed during autopsy may be attributable to treatment-related artifacts.

## Introduction

Diffuse hepatic hemangiomatosis (DHH) is a benign vascular tumor of the liver characterized by replacement of the hepatic parenchyma and an infiltrative border. It primarily occurs in neonates and is extremely rare in adults (1). Histopathological evaluation of DHH is essential for a definitive diagnosis (1), as differential diagnosis remains challenging, even on imaging.

Histologically, it is important to distinguish DHH from hemangiomas and hepatic angiosarcomas. DHH is characterized by dilated vascular spaces and absence of marked cytological atypia, resembling a hemangioma. However, compared to hemangiomas, DHH exhibits an infiltrative growth pattern. The differentiation from hepatic angiosarcomas relies primarily on the absence of cytological atypia. To the best of our knowledge, only one report has described specific genetic mutations or molecular abnormalities associated with DHH (2); therefore, the diagnosis is primarily based on clinical presentation and histopathological features.

The incidence and mortality rates of adult DHH remain unclear because of its rarity. Although many cases follow an indolent course similar to that of hepatic hemangioma, several reports have described rapid progression with diffuse involvement of the entire liver, leading to deterioration of the general condition and death, thereby deviating from the typical benign behavior. Therefore, it is important to distinguish between DHH and hepatic hemangiomas. However, when DHH exhibits aggressive clinical behavior, differentiating it from hepatic angiosarcoma can be extremely challenging. The present case was a diagnostically challenging case of DHH that demonstrated a rapid progression within approximately 6 months, ultimately resulting in a fatal outcome and posing difficulty in differentiating it from hepatic angiosarcoma. In addition, pleomorphic endothelial cells, which are not typically observed, were identified during autopsy, warranting careful histopathological evaluation.

## Case description

**FIGURE 1:**
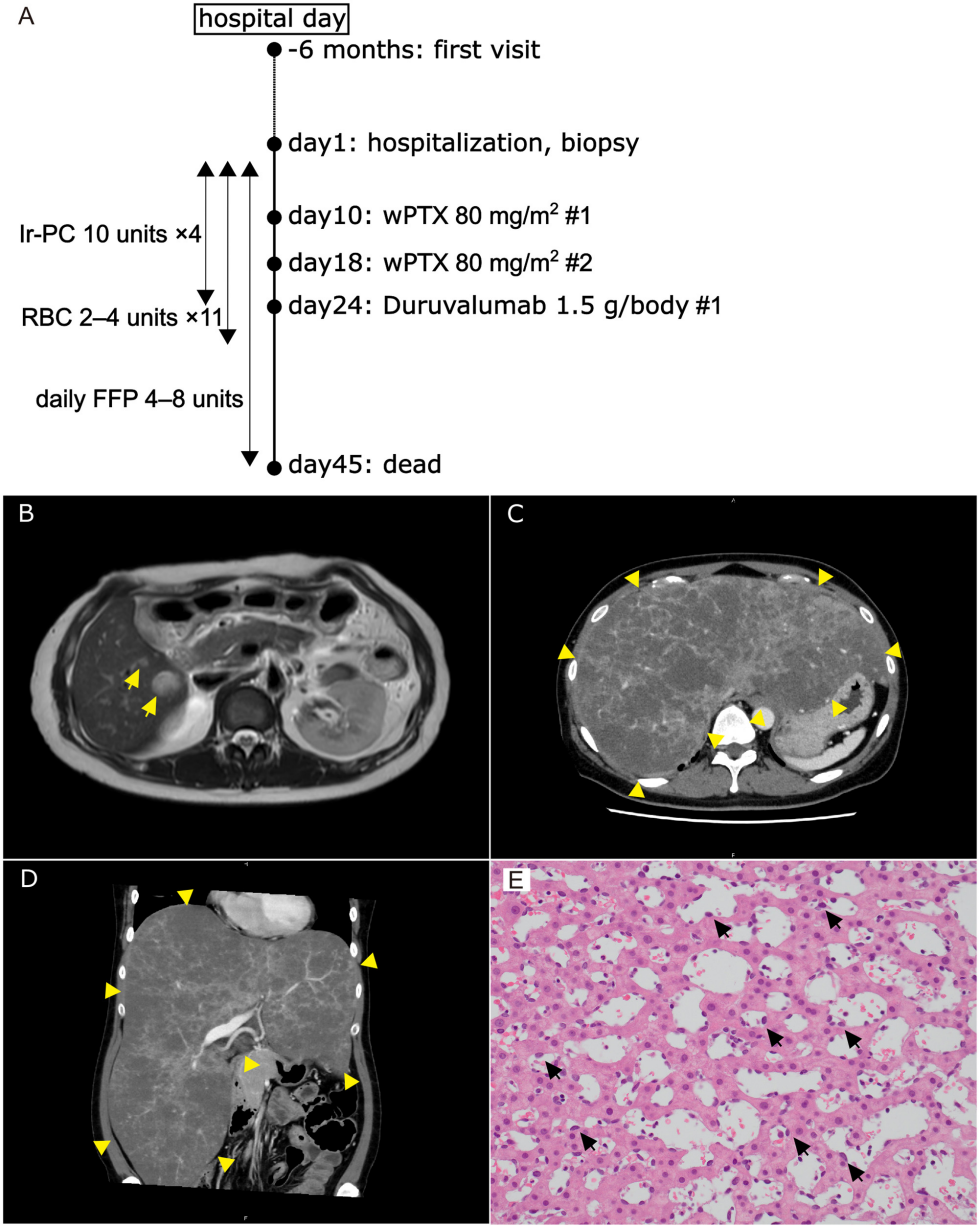
Radiology imaging and biopsy findings of the lesion. **(A)** Timeline of the clinical course of the patient, including percutaneous liver biopsy and treatments. **(B)** An initial abdominal MRI reveals multiple T2-hyperintense lesions. This slice shows two lesions (arrows). **(C,D)** Arterial-phase contrast-enhanced computed tomography (CT) performed 6 months after the initial MRI shows multiple low-density areas (32 HU) of varying sizes diffusely involving the entire liver in the axial [**(C)**; maximum liver diameter, 272 mm] and coronal[**(D)**; maximum liver diameter, 347 mm] planes (arrowheads). **(E)** Biopsy findings show dilated sinusoids lined by enlarged endothelial cells with minimal atypia (arrows). wPTX, weekly paclitaxel; Ir-PC, irradiated platelet concentrate; RBC, red blood cell; FFP, fresh frozen plasma.

A 51-year-old female was referred to our hospital with primary biliary cholangitis (PBC). She reported a history of smoking and alcohol consumption. At that time, an abdominal MRI revealed multiple T2-hyperintense lesions measuring up to 2 cm in diameter (Figures 1A, B) in the normal-sized liver (maximum liver diameter 22 cm), raising suspicion of a hemangioma. The patient was followed up conservatively; however, 6 months later, she developed sudden abdominal distension. Computed tomography (CT) performed at that time revealed hepatomegaly with liver enlargement up to 34 cm in the craniocaudal diameter. Numerous diffusely distributed low-density areas (LDAs; 25 HU) were observed throughout the liver. On arterial-phase contrast-enhanced CT, these LDAs remained poorly enhanced (32 HU), but showed evidence of neovascularization (Figures 1C, D). These findings suggested rapid progression of the previously noted multiple lesions and raised concerns about hepatic angiosarcoma (3). She was admitted for further evaluation. On admission, the laboratory findings demonstrated anemia (hemoglobin, 7.8 g/dL), thrombocytopenia (platelet count, 5.9 × 10^4^/L), prolonged prothrombin time (PT-INR 1.35), elevated D-dimer levels (120.1 μg/mL), and decreased fibrinogen levels (59 mg/dL), consistent with disseminated intravascular coagulation (DIC). DIC was considered multifactorial, likely related to consumptive coagulopathy associated with hepatic lesions, with possible contribution from impaired hepatic dysfunction (AST, 39 U/L; ALT, 55 U/L; LDH, 134 U/L; total bilirubin, 0.71 mg/dL; albumin, 3.4 g/dL). Thus, she required four irradiated platelet concentrate (Ir-PC) transfusions, 11 red blood cell (RBC) transfusions, and daily fresh frozen plasma (FFP) transfusions. No clinical evidence of infection was observed during hospital stay.

A percutaneous liver biopsy was performed on hospital day 1. Histopathological examination of the biopsy specimen revealed dilated sinusoids with hypertrophic lining cells; however, cellular pleomorphism was not prominent (Figure 1E). Immunohistochemical staining was positive for endothelial cell markers, including CD31, ETS-related genes (ERG), and CD34. Assessment of the Ki-67 labeling index is challenging because of the difficulty in distinguishing endothelial cells from non-neoplastic background cells, which results in an unreliable evaluation. Based on these findings, the patient was tentatively diagnosed with a benign vascular neoplasm.

Nevertheless, her abdominal pain worsened, and the tumor size continued to enlarge (39 cm craniocaudal liver diameter on hospital day 11). These clinical features suggest that biopsy specimens may not adequately reflect the overall tumor histology. Therefore, a hepatic angiosarcoma was suspected. Tumor resection was deemed unfeasible because of the extent of the disease and the patient’s general condition. She received weekly paclitaxel (wPTX, 80 mg/m^2^) twice, followed by a single dose of durvalumab (1.5 g/body); however, no clinical improvement was observed. On hospital day 45, the patient remained alert and oriented until the onset of sudden clinical deterioration, after which she became unresponsive and died. Clinically, hepatic failure was considered the most likely cause of death. Subsequently, an autopsy was performed.

**FIGURE 2:**
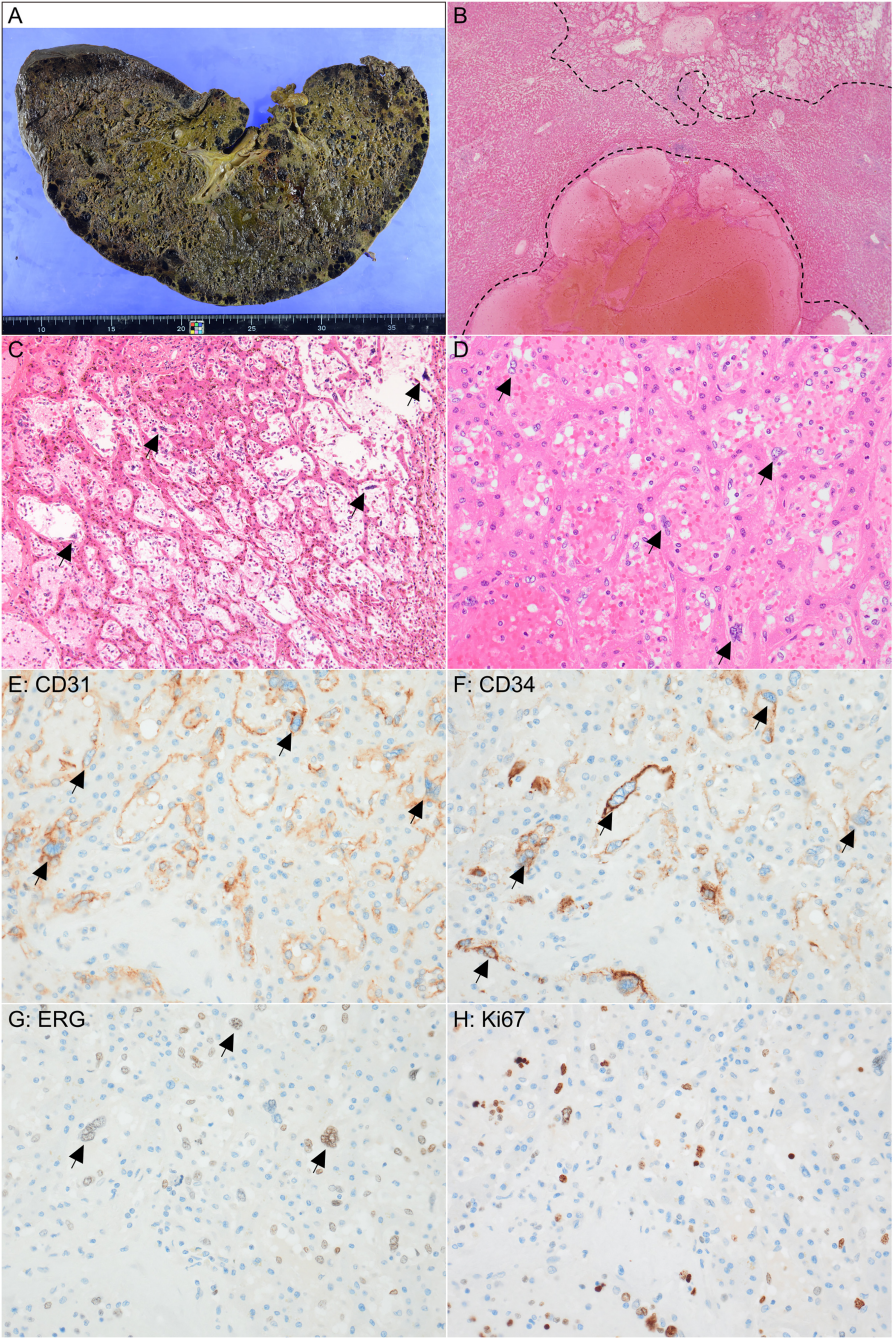
Autopsy findings. **(A)** Cut surface of the formalin-fixed resected liver. The liver appears markedly enlarged and multilocular. **(B)** Low-power microscopic view shows variably dilated vessels forming cystic spaces with ill-defined boundaries (dashed line) (hematoxylin-eosin [H&E] staining; Original magnification ×20). **(C,D)** Magnified microscopic view shows atrophic intervening hepatocytes and thin fibrotic septa between the dilated vessels. The vessels are lined by a single layer of flattened endothelial cells with enlarged and pleomorphic nuclei (arrows) [H&E staining; **(C)**, Original magnification ×100; **(D)**, Original magnification ×200]. **(E–G)** Immunostaining of endothelial cells highlights the pleomorphic lining cells (arrows) [Original magnification ×200; **(E)**, CD31; **(F)**, CD34; **(G)**, ERG]. **(H)** Endothelial cells showing a slight increase in Ki-67 positive cells (Ki-67 immunostaining; Original magnification ×200).

The autopsy revealed a large amount of hemorrhagic ascites in the abdominal cavity. The liver was markedly enlarged, weighing 5,200 g and measuring 26.5 cm in diameter. The liver surface appeared fragile with extensive hemorrhagic fluid exuding from the parenchyma. The liver surface appeared multicystic and the cut surface revealed multiloculated cystic structures (Figure 2A). Vascular lesions were not identified in any other organs. Based on these findings, the cause of death was hypovolemic shock due to a hemorrhage associated with tumor rupture.

Histologically, multiloculated cystic areas in the liver were composed of variably dilated vascular spaces with poorly defined boundaries (Figure 2B). The intervening hepatocytes were atrophic and the vascular walls exhibited varying degrees of fibrosis (Figures 2C, D). The dilated vascular channels were lined with a single layer of flattened endothelial cells with nuclear enlargement. Some cells exhibited pleomorphisms. No histological heterogeneity was observed among the lesions. Immunohistochemically, the cells were positive for CD31 (Figure 2E), CD34 (Figure 2F), and ERG (Figure 2G), indicating a vascular endothelial origin. The Ki-67 labeling index was 10%–20%, indicating a slight increase (Figure 2H). Accordingly, the lesion was diagnosed as a DHH. Evaluation of p53 expression was inconclusive owing to staining artifacts.

## Discussion

Diffuse hepatic hemangiomatosis in adults is a rare, benign vascular tumor of the liver without notable cytologic atypia, resembling a hemangioma but exhibiting an infiltrative growth pattern. Its natural history remains unclear owing to its varied prognosis. Previously reported cases have shown a slight female predominance (11 men, 18 women), with a wide age range at onset (mean 51.8 years, range 22–83 years). Most patients either did not receive treatment or underwent surgical resection, and over half of them survived (Table 1) (1–29). Rapid and progressive fatal outcomes, as observed in the present case, are uncommon. An association between oral contraceptive use and metoclopramide exposure has also been suggested (1). However, the present patient had no history of exposure. Although PBC was present in this patient, no pathogenic association between PBC and DHH or its acute enlargement was established.

**TABLE 1:** Summary of the 30 reported cases of adult-onset diffuse hepatic hemangiomatosis (1–29).

Characteristic		Value
Age (years), mean (range)		51.8 (22–83)
Male/Female (*n*)		11/18 (N/A = 1)
Single node (*n*; largest diameter)		3 (324 mm, 200 mm, N/A)
Multiple or diffuse (*n*)		24 (N/A = 3)
Tumor largest diameter (mm), mean (range)		96.5 (10–324)
Therapy (*n*)	No treatment	11
	Resection	5
	Transplantation	1
	TAE + transplantation	2
	Other	7
	N/A	4
Outcome (*n*)	Alive	18
	Dead	8
	N/A	4

TAE, transcatheter arterial embolization; N/A, not available.

Histologically, DHH is characterized by thin-walled, dilated vascular spaces that replace the surrounding hepatic parenchyma and are often accompanied by fibrous septa. These vascular channels are lined with a single layer of endothelial cells, and the lesion shows an infiltrative border (1). It can be distinguished from hepatic angiosarcoma by the absence of cytological atypia and histological heterogeneity, which typically ranges from well-differentiated to poorly differentiated areas within the same tumor. In the present case, the biopsy specimen exhibited features suggestive of a benign tumor. However, given the rapid tumor growth and deterioration of the patient’s general condition, the biopsy specimen was considered insufficient as it may not have included the malignant component. Histological examination of the autopsy specimen revealed notable cytological atypia necessitating differentiation from hepatic angiosarcoma. Nonetheless, the absence of histopathological heterogeneity, lack of metastasis, and low Ki-67 labeling index supported the final diagnosis of DHH. The appearance of pleomorphic cells and fibrotic areas has been interpreted to be within the range of chemotherapy-induced fibrosis.

There are currently no established guidelines or standard treatment strategies for DHH. Given the rapid progression observed in some cases, therapeutic strategies beyond surgical resection have occasionally been explored. However, evidence regarding the efficacy of chemotherapy for DHH remains limited, with only two cases reported to date (Table 2). In these reports, patients were treated with a combination of thalidomide and sirolimus (2) or with an anti-VEGF inhibitor (4). The former patient survived, whereas the latter underwent postmortem evaluation during which no pleomorphic cells were observed, unlike in the present case. Both of the treatments were initiated for refractory Kasabach–Merritt syndrome. In contrast, the agents used in our case, PTX, a microtubule-stabilizing agent that induces mitotic arrest, and durvalumab, a PD-L1–blocking antibody, exerted direct cytotoxic or immune-mediated effects on tumor cells. This mechanistic difference may have contributed to the emergence of atypical endothelial cells observed in this case, given that PTX has been reported to induce nuclear abnormalities in endothelial cells (30). Furthermore, both wPTX and durvalumab exhibited limited efficacy. This limited response may be attributable to the lower mitotic activity of endothelial cells compared to that of hepatic angiosarcoma, which likely reduces the cytotoxic effect of wPTX. In addition, DHH may be characterized by low immunogenicity and limited immune cell infiltration, which could have contributed to the limited response to durvalumab. Histopathological examination revealed sparse infiltration of immune cells within the lesion.

**TABLE 2:** Adult-onset diffuse hepatic hemangiomatosis cases treated with chemotherapy or with fatal outcomes.

References	Age	Sex	Distribution pattern	Tumor size (cm)	Extrahepatic lesion	Therapy	Outcome	Cause of death
He et al. (2)	59	F	Multiple	N/A	Spleen	PSL, 60 mg/day (reduced gradually after 3 weeks); thalidomide, 100 mg/day; and sirolimus, 1 mg/day	Alive (4 months)	–
Shimizu et al. (4)	83	F	Diffuse	N/A	Spleen	Symptomatic treatment and VEGF inhibitor	Dead	DIC
Militz et al. (5)	59	M	N/A	N/A	N/A	No	Dead	Liver failure
Supakul et al. (6)	53	M	Multiple	N/A	N/A	No	Dead	Heart failure
Jun and Yun (7) .	69	F	Multiple	max 2.7 × 2.0	N/A	No	Dead	Bleeding
Kim et al. (8)	33	F	Multiple	N/A	No	No	Dead	Liver failure
Langner et al. (9)	62	M	Multiple	6 × 4 × 3.5	Spleen	N/A	Dead	Liver failure
Maeda et al. (10)	30	F	Multiple	2.5–5	Spleen	Splenectomy, radiotherapy	Dead	Peripheral vascular collapse
Kane and Newman (11)	67	F	Multiple	N/A	Bone, pleura	PSL	Dead	N/A
Present case	51	F	Diffuse	26.5	No	Chemotherapy	Dead	Bleeding

M, male; F, female; N/A, not available; PSL, prednisolone; DIC, disseminated intravascular coagulation.

To date, eight fatal cases of DHH have been documented (Table 2). All the cases involved multiple or diffuse hepatic lesions that were not amenable to surgical resection. There is no clear cutoff value for lesion size; however, hepatic failure, bleeding, and DIC are the most common features associated with fatal outcomes. In the present case, diffuse hepatic involvement, rapid progression, DIC development, and progressive hepatic dysfunction were also observed, all of which are recognized as high-risk features for mortality. These cases, including the present case, which show rapid progression and fatal outcomes, may deviate from those of typical benign tumors. In this context, a new disease concept, hepatic small-vessel neoplasm (HSVN), has been proposed to reflect the biological aggressiveness of such lesions.

Recent molecular pathology studies have demonstrated frequent mutations in GNAQ and GNA14 in HSVN (31), implicating these alterations in vascular differentiation. In our case, although genetic analyses of both the biopsy and autopsy specimens were attempted, no actionable mutations were identified. This may have been due to the low cellularity of the biopsy specimen, which resulted from its small size and the sinusoidal distribution of endothelial cells. In addition, the autopsy specimens were not well-preserved because of postmortem tissue degradation. This suggests that the preparation of larger, well-preserved tissue specimens may be necessary to improve the diagnostic yield of molecular analyses in such cases. Further accumulation of cases and comprehensive molecular analyses are warranted to elucidate the biological relationships and distinctions between DHH and HSVN.

## Conclusion

Here, we report a rare case of adult-onset DHH that exhibited rapid progression and ultimately resulted in a fatal outcome, with an unusual clinical course characterized by ineffective chemotherapy. Cytological atypia was a notable autopsy finding. Given its aggressive clinical course, careful differentiation from hepatic angiosarcoma is of critical diagnostic value. Although the presence of pleomorphic endothelial cells may complicate this distinction, these features should be interpreted with caution as they may represent treatment-related artifacts.

## Data Availability

The original contributions presented in this study are included in the article/supplementary material, further inquiries can be directed to the corresponding author.
